# *duet*: An R package for dyadic analysis of motion data generated by OpenPose

**DOI:** 10.3758/s13428-025-02817-w

**Published:** 2025-09-24

**Authors:** Themis N. Efthimiou, Catherine J. Crompton

**Affiliations:** Centre for Clinical Brain Sciences, University of Edinburgh, Edinburgh, UK

**Keywords:** Nonverbal communication, Video coding, Naturalistic communication, Dyadic interaction analysis, Kinematic analysis

## Abstract

Research into dyadic social interactions has expanded significantly, enabling a deeper understanding of the dynamic processes underlying interpersonal communication. As the use of larger datasets becomes increasingly common in this field, the need for scalable and efficient analytical tools has grown. Automated coding methods, such as those provided by OpenPose, an open-source software for detecting and tracking human motion, offer significant advantages for analysing the movement of two individuals during an interaction. However, the processing and analysis of large quantities of JSON output files generated by OpenPose remain a considerable challenge. To address this, we introduce *duet*, an R package designed to streamline the processing and analysis of OpenPose output data, particularly in the context of dyadic interactions. The package provides a suite of functions for data cleaning, interpolation, kinematic analysis, and visualisation, offering researchers a comprehensive and user-friendly workflow. By simplifying the handling of OpenPose data, *duet* aims to facilitate large-scale, automated analysis of dyadic social interactions, with minimal coding experience, thereby advancing methodological capabilities in social and behavioural sciences.

## Introduction

The use of video recording as a research tool in psychology can be traced back to the pioneering work of René A. Spitz. Through his groundbreaking studies on infants, documented in publications exploring hospitalism and anaclitic depression and brought vividly to life in his film *Grief: A Peril in Infancy* (Spitz, 1950; Spitz & Wolf, 1946), Spitz demonstrated how moving images could capture the subtle dynamics of early emotional and social development, laying the groundwork for subsequent generations of video-based observational research. This early use of video recording as a research tool set the stage for its subsequent integration into various psychological subfields. Developmental psychologists expanded Spitz’s methods to systematically observe parent–infant (Kemppinen et al., 2005) and peer interactions (Leclère et al., 2016; McNaughton et al., 2024), refining their understanding of socialisation and learning processes. Clinical psychologists employed video to document therapy sessions and assess diagnostic interviews, enabling a more nuanced exploration of client behaviours, therapist techniques, and treatment outcomes (Ramseyer, 2020a; Ramseyer & Tschacher, 2011; Warnock-Parkes et al., 2017). In social psychology, video recordings facilitate the study of group dynamics, non-verbal communication, and interpersonal relationships (Dunbar et al., 2023; Foster et al., 2024; Fujiwara et al., 2020; Lin et al., 2023). At the same time, lab-based experiments can be used to ensure participants follow the required conditions for each task (Efthimiou et al., 2024a, 2024b; Wagenmakers et al., 2016).

Historically, generating quantifiable data from the videos relied heavily on manual coding and frame-by-frame examination, requiring extensive training to categorise behaviours systematically. For example, mastering the Facial Action Coding System (FACS) can require over 100 h of training (Ekman & Friesen, 1978). Although these methods yield valuable insights, they are time-consuming and labour-intensive, often requiring up to ten times the length of the source video, thus limiting scalability. The reliance on human observation also introduced subjective biases and inconsistencies (Burgess et al., 2023; Chouinard et al., 2019). An alternative option to video recordings—and the limitations of manual coding—is to use specialised equipment such as motion capture equipment (e.g., OptiTrack, Vicon, Kinect). This equipment has been used extensively to assess movement kinematics (Cook et al., 2013), the effects of live performance versus recorded music on audience head movement (Swarbrick et al., 2019), and sign language (Puupponen et al., 2015). This technology, however, can be costly. It may also be unsuitable for certain populations, as infants might resist wearing the markers (Chouinard et al., 2019), and neurodivergent participants often benefit from less intrusive methods (Plunk et al., 2023).

A more cost-efficient and easily employed method of analysing movement is automated video coding (Congdon et al., 2018; Dunbar et al., 2022; Haggerty & Hilsenroth, 2011). Breakthroughs in computer science and innovative analysis techniques have enabled the development of tools to automate video analysis (for review, see Dunbar et al., 2022; Gregori et al., 2023). This includes frame-differencing methods (Bobick & Davis, 2001; Ramseyer, 2020b), which detect changes between consecutive video frames to identify motion. For more complex feature extraction directly from video frames, convolutional neural networks (CNNs) are often utilised. CNNs are excellent for extracting spatial features, while recurrent neural networks (RNNs) and long short-term memory (LSTM) networks can subsequently capture temporal dependencies from these features, making them ideal for analysing sequences of actions or behaviours over time. Further, they can be used in real time or retroactively. These methods have given rise to a variety of open-source programmes, including Py-feat (Cheong et al., 2023), OpenFace (Baltrušaitis et al., 2016), OpenPose (Cao et al., 2019), Opensmile (Eyben et al., 2010), LibreFace (Chang et al., 2024), DeepLabCut (Mathis et al., 2018), Motion Energy Analysis (Ramseyer, 2020b), and commercial software (FaceReader; iMotions). These tools have not only reduced the time and effort required for video analysis but have also enabled researchers to tackle larger datasets and uncover patterns that were previously undetectable. This evolution in methodology has significantly enhanced the scope and accuracy of psychological research, opening new avenues for understanding human behaviour in both naturalistic and experimental settings.

Indeed, research increasingly focuses on social interactions within their natural settings and the bidirectional influence that individuals in a dyad have on one another (Kenny & Kashy, 2011; Malloy & and Albright, 2001). This principle of dyadic interdependence means that one person’s actions influence the other’s immediate response, which in turn shapes the first person’s next action, creating a continuous feedback loop. Such research explores a wide range of dynamic behaviours. For instance, joint-action studies examine how individuals seamlessly integrate their actions to achieve common goals, such as lifting an object together (Azaad & Sebanz, 2025; Grinspun et al., 2024; Sander et al., 2025). Other work delves into the intricate synchrony and non-verbal interplay essential for coordinated performances by duet musicians (Bishop, 2023; Cross et al., 2024; Dotov et al., 2021; Hartmann et al., 2019; Orgs et al., 2024), the verbal and non-verbal exchanges that shape dyadic communication (Bohy et al., 2022; Efthimiou et al., 2025a; Hamilton & Holler, 2023; Jokinen et al., 2016), and the impact of matched or mismatched neurotypes on information transfer and rapport (Crompton et al., 2020, 2025; Rifai et al., 2022). A related body of work evaluates how the behavioural (or motor) synchronisation between dyadic partners influences social outcomes such as bonding (Efthimiou et al., 2025b; Lakens & Stel, 2011) and altruistic decision-making (Fujiwara et al., 2022).

However, analysing the data produced by these automated tools is not always straightforward, especially with the large quantities of time-series data generated. Here we focus on the open-source software OpenPose, which generates two-dimensional (2D) coordinates of joint locations (i.e., elbow, shoulder) per frame. This software is recognised as one of the leading pose estimation tools (Ino et al., 2024; Li et al., 2021; Yamamoto et al., 2021) used in sports science (Rabin et al., 2018), sign language (Trettenbrein & Zaccarella, 2021), and emotion categorisation (Försterling et al., 2024). Further, there has been growing use in dyadic research given the ability of OpenPose to track two people in a frame, being used to assess synchronisation between dyads (Fujiwara et al., 2020) and kinematic measures (Efthimiou et al., 2025a). While powerful, its application to dyadic interactions, encompassing how pairs of individuals coordinate, communicate, and influence each other, presents notable challenges.

OpenPose assigns identifiers to detected people in each frame independently, which can result in inconsistent labelling across frames, making it difficult to analyse continuous interactions between the same two individuals, as there is no straightforward way to Link their movements throughout the video. As a result, studying interpersonal dynamics such as synchronisation, mimicry, or joint attention becomes complicated. Further, OpenPose generates a JSON file per frame, one for each frame of video, which can be cumbersome to handle due to their nested structure and the sheer volume of files; for example, a 5-min video at 30 frames per second (FPS) produces 9,000 files. Managing thousands of JSON files makes data aggregation and preprocessing a daunting task, particularly for researchers who may not have advanced programming skills, and this complexity can hinder the efficient analysis of movement data extracted from videos. A final Limitation arises during data analysis. Once the files are aggregated, the 2D coordinates produce a wide data format, typically consisting of *x* and *y* dimensions for 24 body parts—a figure that increases significantly if hands (42 keypoints) or the face (70 keypoints) are included. Transforming this complex, raw data into meaningful insights requires coding experience that not all researchers possess, which in turn limits the accessibility and use of the technology. While an R package is available for analysing OpenPose data to bridge some of these limitations (Trettenbrein & Zaccarella, 2021), its development was primarily driven by the need to create an advanced method for controlling and quantifying bodily motion in video clips of individuals signing or gesturing from a frontal view. Consequently, its primary design focuses on the analysis of single-person recordings, often for stimulus control in research fields like sign language or gesture studies.

Therefore, we introduce *duet*, a user-friendly, free, and open-source R package explicitly designed for the analysis of OpenPose data from dyadic interactions, distinguishing it from tools primarily focused on single-subject analysis for purposes like stimulus control. *duet* (Efthimiou, 2025) offers a suite of features tailored to the complexities of two-person data. It automates the process of reading thousands of JSON files and consolidates them into a single CSV file, simplifying data handling and making it more accessible for manipulation using standard data analysis techniques. Crucially, it includes two methods to identify and consistently track each person in the video. One can use the OpenPose algorithm tracker or, based on their spatial location and movement patterns, maintain consistent identifiers for each individual across all frames to ensure accurate relational analysis of their interactions over time.

To enhance data quality and prepare for robust analysis, *duet* provides functions for smoothing raw coordinate data to reduce noise inherent in video capture and pose estimation. It further offers tools for visualising algorithm confidence ratings and the missingness of data, generating descriptive summaries of the time series (e.g., mean, median, interquartile range [IQR], skewness, variance, kurtosis) and implementing various methods for the removal or handling of unreliable data points. For analytical depth, duet calculates essential kinematic variables such as velocity, acceleration, and jerkiness of movement. Significantly, these can be computed not only for individuals but also in a manner that enables comparative and relational analysis between dyadic partners. Beyond these kinematics, duet incorporates advanced analytical capabilities such as functions to compute motion energy (Fujiwara & Yokomitsu, 2021; Ramseyer, 2020b) and wavelet coherence (Dunbar et al., 2022; Fujiwara & Yokomitsu, 2021; Fujiwara et al., 2022), facilitating the direct quantification of interpersonal synchrony and other dynamic relational aspects. Visualisation is a core component, with capabilities to plot movement trajectories and single static frames, aiding in the qualitative assessment of behavioural patterns. It can also re-animate the tracked movements as point-light displays (Johansson, 1973) specifically for dyads, which are simplified representations of motion using points of light at key joints. These animations can be exported as MP4 videos, allowing researchers to visually inspect and present the dynamic aspects of the interactions. Furthermore, for integration with other specialised software and analytical workflows, *duet* allows data to be exported in the rMEA package format (Kleinbub & Ramseyer, 2021).

## Methods

### OpenPose

OpenPose (Cao et al., 2017, 2019) can be used through both command-line and graphical interfaces, allowing users to process either static images or videos. After installing the required dependencies (as explained on the official GitHub web page; https://github.com/CMU-Perceptual-Computing-Lab/openpose), one can simply run the provided examples (e.g.,./build/examples/openpose/openpose.bin –image_dir examples/media/) to detect and extract keypoints in images. OpenPose generates outputs in multiple data formats (e.g., JSON) that encapsulate information about keypoints. In this context, keypoints refer to the spatial coordinates of specific anatomical landmarks, such as joints, facial features, and hand positions, that are detected in each frame of an image or video sequence. Each keypoint typically comprises an *x*-coordinate, a *y*-coordinate, and an associated confidence score, which together facilitate a detailed representation of human posture and movement. These structured outputs can be readily incorporated into a variety of workflows. However, when dealing with multi-person scenes, particularly for dyadic analysis, it is important to consider how individuals are identified and tracked across frames. OpenPose’s default for multi-person detection is lightweight but can be susceptible to errors, especially if individuals swap positions or move significantly within the frame. For more robust dyadic tracking directly within OpenPose, a pragmatic solution is to utilise its built-in ‘tracking’ option. For instance, using command-line flags such as ‘–tracking 1 –number_people_max 2 –tracking_max_age 30’ can help propagate identities across frames (e.g., for up to 30 frames if a person is temporarily lost), making the tracking of two individuals more consistent. For even more advanced tracking capabilities, particularly in complex scenarios, other approaches such as integrating DeepSORT with OpenPose (as demonstrated in projects like https://github.com/ortegatron/liveposetracker) warrant consideration, offering more sophisticated algorithms for maintaining persistent identities over time.

### Package design and core functionalities

The *duet* package consists of several functions (see Table 1). Notably, all function names begin with the prefix ‘op’, signifying that these functions are specifically designed to process data structured in the OpenPose format. This naming convention ensures clarity and modularity in the codebase, making it immediately apparent which functions are tailored for OpenPose outputs. This design choice anticipates future developments of *duet*, which aim to integrate additional open-source tools, such as OpenFace (Baltrušaitis et al., 2016), that utilise distinct file structures. The framework allows seamless expansion by maintaining function prefixes tied to specific data formats, ensuring compatibility with diverse packages (Table 2).

**Table 1:** A comparison of features between the OpenPoseR and *duet* software packages, highlighting their respective focus on single-person and dyadic interaction analysis

Feature	OpenPoseR	*duet*
Primary design focus	Single-person recordings	Single and dyadic interactions
Kinematics	Primarily single-person	Single and dyadic
Motion energy calculation	No	Yes
Wavelet coherence	No	Yes
Data quality plots	No	Yes
Descriptive summary	No	Yes
Bad data removal	No	Yes
rMEA export	No	Yes
Time-series smoothing	No	Yes
Visualisation	Basic	Advanced
Dyadic-specific metrics	No	Core focus

**Table 2:** List and description of all available functions contained within the package

Function	Description
op_animate_dyad	Generates animations for dyadic interactions based on motion data
op_apply_keypoint_labels	Applies labels to keypoints extracted from OpenPose data
op_batch_create_csv	Creates CSV files in batches for keypoint data processing
op_compute_acceleration	Computes acceleration from position or velocity data
op_compute_euclidean_distance	Calculates Euclidean distance between specified keypoints
op_compute_jerk	Computes jerk (rate of change of acceleration) from data
op_compute_velocity	Calculates velocity from position data
op_create_csv	Creates individual CSV files for each participant or dyad
op_interpolate	Interpolates missing data points in time series
op_merge_dyads	Merges data for all dyads into a single file
op_plot_openpose	Plots data from OpenPose keypoint tracking
op_plot_quality	Visualizes the quality of motion capture data
op_plot_timeseries	Plots time-series data for analysis or visualization
op_remove_keypoints	Removes specified keypoints or undetected data from datasets
op_smooth_timeseries	Smooths time-series data to reduce noise
op_summarise()	Calculates descriptive statistics for time series
op_compute_motionenergy()	Computes a motion energy time series from keypoint displacement
op_compute_coherence	Computes cross-wavelet coherence of dyadic motion energy

The main function of the package is op_create_csv(), which processes 2D pose estimation data from JSON files and converts it into structured CSV files for further analysis.

**Fig. 1:**
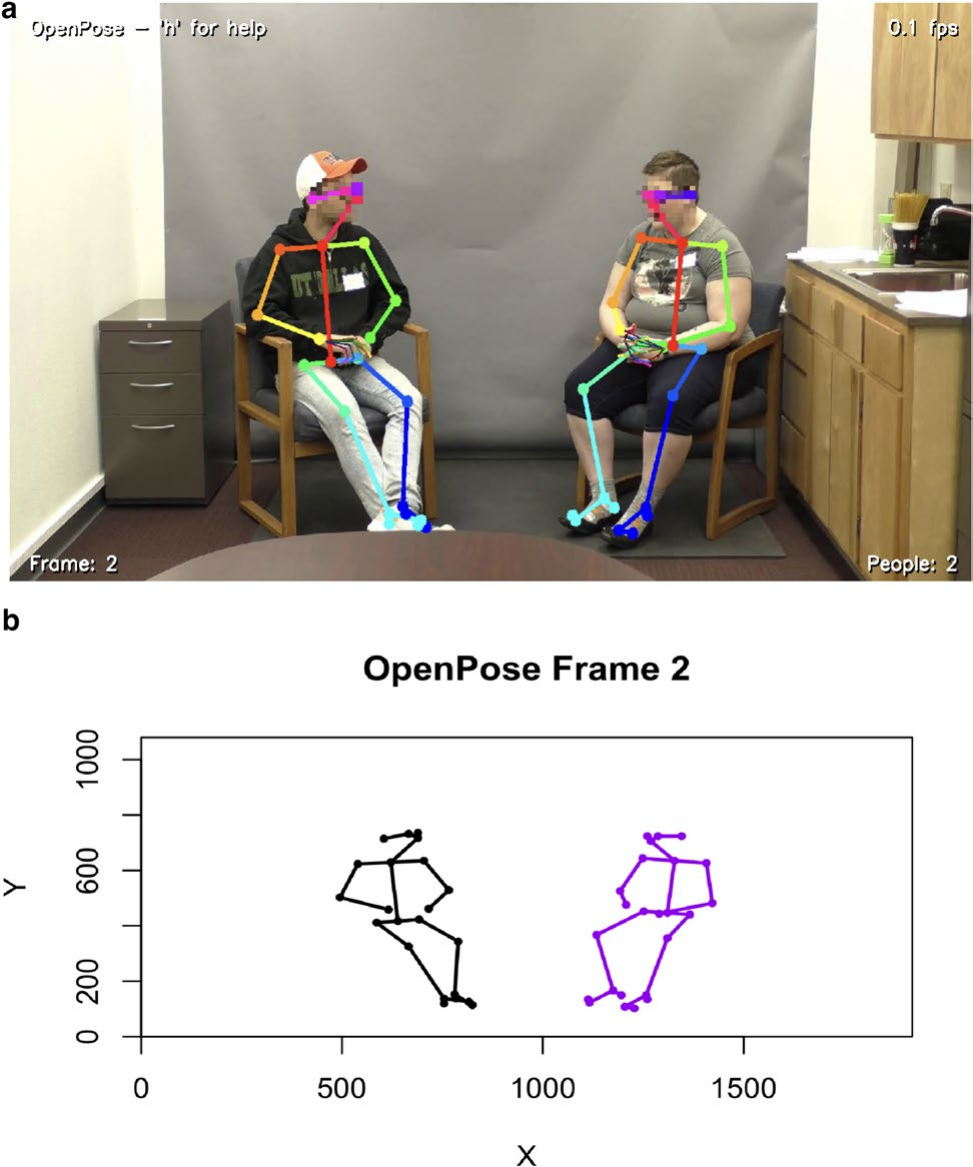
Visualisation of dyadic interaction captured by OpenPose and reconstructed PLDs. Note: Panel **a** shows a screenshot of the OpenPose analysis windows, specifically frame two of the two individuals seated and their skeletal motion features overlaid in colour-coded keypoints and connections. The faces are pixelated for anonymity; this is independent of OpenPose. Panel **b** depicts the corresponding reconstructed PLD representations of the individuals, plotted using the op_openpose() function

One method for dyad identification within this function relies on participants remaining in relatively fixed positions throughout the recording, ensuring that neither crosses into their partner’s designated screen region (e.g., left and right sides; see Fig. 1). This split-screen approach, common in many controlled experimental setups (Efthimiou et al., 2025b; Georgescu et al., 2020; Glass & Yuill, 2024; Ramseyer, 2020b; Tschacher et al., 2014; Zhao et al., 2022), assumes individuals do not swap sides. However, this method has clear limitations in scenarios with more dynamic movement or where participants might cross over, as it can lead to incorrect individual labelling.

To provide greater flexibility and address the limitations of the screen-split method, the function includes an optional parameter, use_openpose_order. When use_openpose_order is set to TRUE, *duet* will utilise the person indexing directly provided by OpenPose within the JSON files. This leverages OpenPose’s internal multi-person tracking capabilities, which can be more robust in situations where individuals might move more freely or swap positions, offering users a choice of labelling strategy best suited to their specific video data and experimental design. Regardless of the method chosen, the function consolidates the data, simplifying it for easier manipulation using standard data analysis techniques.

The function ‘op_read_csv()’ reads the JSON files from a specified directory and saves the output to another directory, with options to include data for the body, hands, face, or all three regions simultaneously. The function supports two export formats: ‘individual’, which generates separate CSV files for each person in the scene, and ‘dyad’, which combines data from two individuals into a single file. It identifies the left and right individuals in the frame based on their mean *x*-coordinates relative to the screen’s centre, calculated using the frame_width parameter (default is 1,920 pixels and should be adjusted to the specific video width). Keypoints are extracted and renamed dynamically (e.g., × 0, *y*0, *c*0 for body keypoints), and optionally with labels or filenames in the output. The processed data is saved in CSV format, organised by region (body, hands, or face) and person (left or right), allowing for flexibility in analysing pose data from dyadic interactions. The data is stored in a CSV file and can then be analysed separately in the user’s preferred software or remain within the package for continuous processing and integration into further workflows.

A novel function within the package is the ability to regenerate the video as point-light displays (PLDs) (Johansson, 1973), in the form of either static images or fully compiled videos, thereby isolating motion cues from other visual features. The op_animate_dyad() function generates a video from OpenPose output with options to highlight or connect specific joints, customise each member of the dyad (e.g., specifying different colours), set the frame rate, and extract specific time segments. Adjusting the frame rate allows for the manipulation of perceived velocity, as increasing the frames per second (FPS) speeds up motion, while decreasing it slows movement down. The function compiles individual frames via FFmpeg into a single video, ensuring minimal extraneous detail and thus affording flexibility for applications such as studying biological motion recognition and social perception (Calvo-Merino et al., 2010; Plank et al., 2023).

A companion function, op_plot_openpose(), creates static visualisations of individual frames. This can help identify problematic data segments or demonstrate specific poses without revealing sensitive identifying features. Together, these capabilities provide a convenient way to produce anonymised motion stimuli or investigate fine-grained movement patterns in dyadic interactions. By focusing on motion cues, researchers can examine perception and interaction processes free from potential confounds introduced by other visual characteristics.

### Installation and setup

To begin using *duet*, you will need a current version of R (R Core Team, 2023) (4.0.0 or later is recommended) installed; instructions for doing so can be found on the Comprehensive R Archive Network (CRAN). Once R is set up, *duet* can be installed directly from CRAN by entering install.packages('duet') in the R console, which will handle all necessary dependencies. After installation, simply load the library *duet* can be incorporated into your analytical workflows.

The following commands will install and load the latest version of the package from CRAN or GitHub:

```R.

# Using CRAN.

install.packages('duet').

library('duet').

```

```R.

# Using CRAN.

install.packages('devtools').

devtools::install_github('ThemisEfth/duet', upgrade = 'never').

```

If the focus is to convert your OpenPose data into videos, you will also need the open-source software FFMPEG (https://www.ffmpeg.org/), which is supported on Mac, Windows, and Linux operating systems.

### Illustrative example

Initially, the package requires JSON files generated by OpenPose, typically stored in a directory for each dyad. Package functions then convert these JSON outputs into CSV files for easier handling. For a single directory (e.g., one dyad), users can specify the input_path for the JSON files and the output_path for the CSV output. The package also accommodates different OpenPose models (body, head, and hands). Although most workflows rely on the body model for efficiency, any combination of these models can be processed. For example:

```R.

# Example for a single dyad.

input_path <—'/path/to/openpose_json/dyad01'.

output_path <—'/path/to/openpose_csv/dyad01_output'.

op_create_csv(

input_path,

output_path,

include_filename = TRUE,

include_labels = FALSE,

model = 'body',

frame_width = 1920,

export_type = 'dyad').

```

In a multiple-dyad workflow, users designate a parent directory containing subfolders for each dyad’s JSON files. The function then scans each subfolder in the input path and produces corresponding CSV files in the output path. This approach facilitates efficient handling and processing of multiple dyads within a single run, avoiding the need for loops by inexperienced coders.

```R.

op_batch_create_csv(

input_base_path = './json',

output_base_path = './dyad',

model = 'body').

```

Once created, the CSV files can be imported into R (e.g., using read.csv()) like any standard data file.

```R.

dataframe <—read.csv('./dyads/dyad_16.csv').

```

**Fig. 2:**
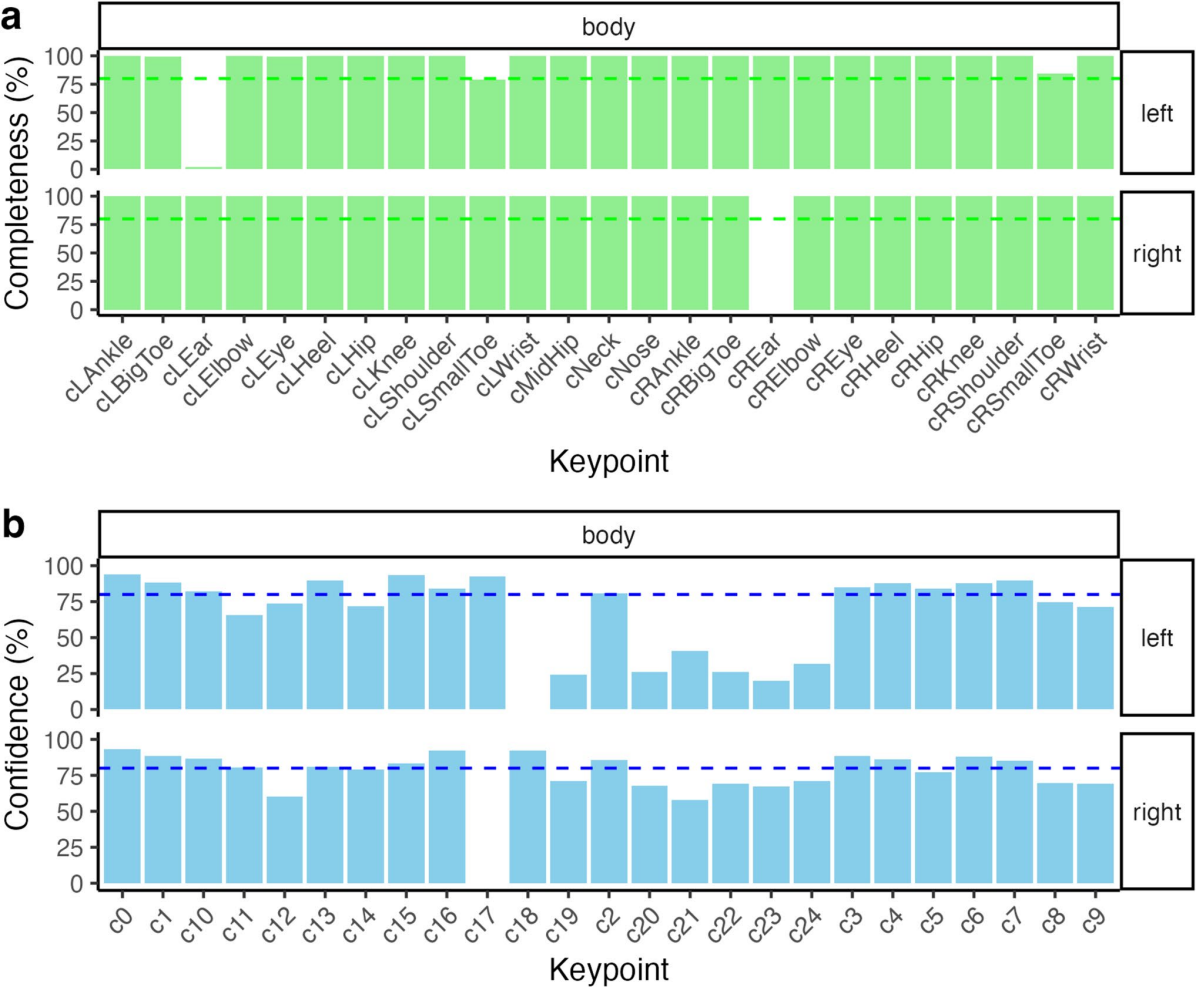
Confidence and completeness of keypoint detection for body movements. *Note*: Panel **a** shows the average confidence levels (%) for each detected keypoint on the body, divided into left and right sides, with dashed lines representing thresholds for filtering. Panel **b** displays the completeness (%) of each keypoint detection, indicating the proportion of frames where the keypoints were successfully detected. Data reflect body keypoint detection performance, highlighting consistency across both confidence and completeness metrics

Each row corresponds to a single video frame, while columns store metadata (e.g., base_filename, frame, region, person) and keypoint coordinates (× 0, *y*0, *c*0, …, × 24, *y*24, *c*24). Here, *x* and *y* denote the spatial coordinates of each detected keypoint, while *c* represents OpenPose’s confidence score. Although numeric labels are sufficient for preprocessing, the op_apply_keypoint_labels() function can replace them with descriptive names for improved interpretability in visualisations (e.g., renaming keypoints as ‘nose’, ‘left eye’, ‘right eye’). This function can be integrated into a pipeline to automatically label keypoints before plotting (see Fig. 2a and b x-axis ticks):

```R.

dataframe |> 

op_apply_keypoint_labels() |> 

op_plot_openpose().

```

At this stage, standard data processing can be performed (e.g., filtering by participant, frame number, or confidence threshold). For illustrative purposes (see Fig. 1), one may generate static plots or videos (e.g., mp4) to evaluate data quality. Further, missing data patterns and confidence scores can be examined across time (see Fig. 2a and b). For instance, a user might impose an 80% completeness criterion for each keypoint, excluding those that fail to meet this threshold. Such steps help refine the dataset and ensure data quality before conducting analyses on dyadic movement.

The op_remove_keypoints() function is designed to clean data by removing unreliable keypoints and their corresponding columns, ensuring consistency between the two members of a dyad. The function processes the data for each unique combination of person (member of the dyad) and region, applying removals either uniformly across both members or independently for each person. It begins by removing any user-specified columns provided via op_remove_specific_keypoints(). If remove_undetected_keypoints = TRUE, the function identifies and removes keypoints where the confidence values are consistently zero, along with their associated *x* and *y* coordinate columns. It then removes keypoints with average confidence values below a specified threshold (op_remove_keypoints_total_confidence()). Additionally, columns with a high proportion of missing or zero values exceeding a user-defined threshold (remove_keypoints_missing_data) are removed. When removals are applied equally across both dyad members, this ensures that the dataset retains a consistent number of frames and keypoints for both individuals, facilitating synchronised analyses. After processing, the function returns the cleaned dataset, ensuring only reliable and synchronized keypoints remain for both members of the dyad.

```R.

df_filtered <—op_remove_keypoints(df,

remove_undetected_keypoints = TRUE,

remove_keypoints_total_confidence =.5,

remove_keypoints_missing_data =.5,

remove_specific_keypoints = NULL,

apply_removal_equally = TRUE).

```

**Fig. 3:**
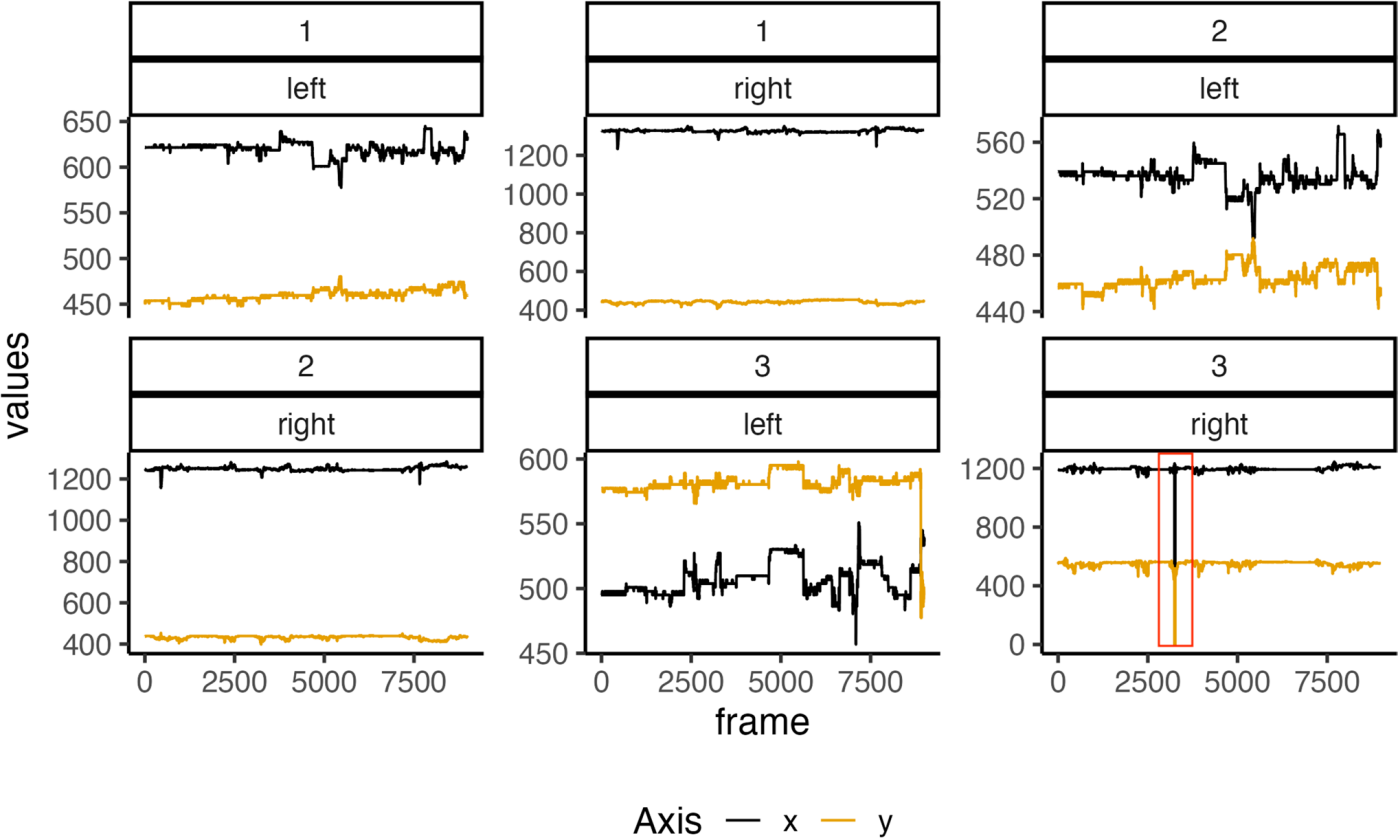
Example of time-series plot. *Note*: The figure illustrates the time series of three keypoints (1, 2, 3) for both members of the dyad, indicated by their seated positions (left or right). For each keypoint, there are two colours: black represents the *x*-coordinate, and yellow represents the *y*-coordinate in 2D space. Drops in the frame, which occur when the algorithm loses track of a keypoint, can be identified by a drop to zero. For example, in the right participant, keypoint 3 is shown in a red box and has a drop to zero

If one wants to interpolate missing data, the op_interpolate() function is designed to handle missing or low-confidence keypoint data in OpenPose outputs by applying cubic spline interpolation to estimate missing values. For instance, in the final panel of Fig. 3, the Line drops to zero before frame 2,500, indicating that no coordinate was detected at that time point (indicated by red outline). If the gap is brief, interpolation using surrounding data can resolve the issue. However, if the dip persists for an extended period, it may be necessary to exclude that time segment from the analysis. The interpolation begins by identifying columns for *x*-coordinates, *y*-coordinates, and confidence scores in the dataset, grouping the data by unique combinations of person and region to process each group independently. For each group, the function loops through all keypoints, identifying rows with low-confidence values (below a user-specified threshold) or missing values, depending on the missing argument. If missing = TRUE, it includes NA values in the interpolation; otherwise, it considers only low-confidence rows. The function performs interpolation using indices of valid (non-missing, above-threshold) data points, ensuring there are at least two valid points for interpolation; otherwise, it skips and issues a warning. The interpolated *x* and *y* values replace the low-confidence or missing entries directly in the dataset, ensuring smooth and plausible trajectories. After processing all groups, the function returns the updated dataset with interpolated values, allowing users to clean and prepare motion data for further analysis or visualisation.

During preprocessing, op_plot_timeseries() can visualise time-series data for either member of the dyad or for specific keypoints (see Fig. 3) by highlighting missing frames, abrupt trajectory changes, or variations in detection confidence.

**Fig. 4:**
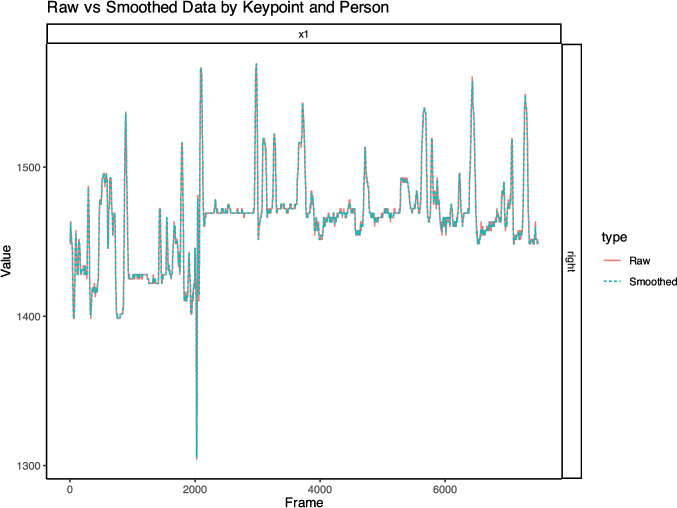
Comparison of raw and smoothed keypoint data across frames. *Note*: The plot illustrates the raw (red line) and smoothed (dashed blue line) data for a single keypoint across frames. The *x*-axis represents the frame number, while the *y*-axis displays the corresponding keypoint value. Smoothing reduces noise and fluctuations observed in the raw data, providing a clearer representation of the keypoint’s trajectory over time

An optional final step involves smoothing time-series data with op_smooth_timeseries(), which offers multiple techniques (e.g., moving average, Kalman–Ziegler adaptive, Savitzky–Golay, and Butterworth). These methods vary in complexity and in how they preserve signal features. For instance, simpler approaches (e.g., moving average) may suffice for low-noise data, while more advanced filters (e.g., Butterworth) are advantageous for noisier recordings. By setting plot = TRUE, users can compare raw and smoothed outputs across both dyad members and all keypoints (see Fig. 4). Incorporating smoothing in the workflow generally helps mitigate artefacts, promoting more accurate downstream analyses such as trajectory modelling.

At this stage, the data is ready for analysis, offering flexibility in analytical choices depending on the research objectives. For example, the dataset can be analysed using various methods to understand dyadic interactions, such as wavelet coherence (Fujiwara et al., 2020) or cross-correlation (Kleinbub & Ramseyer, 2021), to analyse synchronous movement. Additionally, motion energy can be computed by analysing frame-by-frame differences in movement (Efthimiou et al., 2025b; Fujiwara & Yokomitsu, 2021), or using machine learning approaches (Roggio et al., 2024) or several other methods (for review see Gates & Liu, 2016). Currently, the *duet* framework includes support for kinematic analysis, deriving measures such as velocity, acceleration, and jerk (Cook, 2016; Cook et al., 2013; Edey et al., 2017) from the keypoint trajectories.

Velocity, a fundamental kinematic measure, quantifies how quickly a keypoint’s position changes over time. In op_compute_velocity(), it is calculated by comparing consecutive frames and dividing the position change by the time elapsed between them (determined from the video’s frames per second, FPS). Depending on analytic goals, velocity may be computed along each axis (*x* or *y*) or as a single two-dimensional value.

$$v\left[i\right]= \frac{\left(p\left[i\right]- p\left[i-1\right]\right)}{\Delta t}$$

Here,  $p\left[i\right]$  represents the position of the keypoint on a single axis (*x* or *y*) in the current frame, and $p\left[i-1\right]$ is its position in the previous frame. The denominator $\Delta t$ corresponds to the time interval (in seconds) between the two frames. This formulation is particularly useful for analysing linear motion along one dimension, such as vertical or horizontal displacement alone.

When studying overall movement in two dimensions, the Euclidean distance between consecutive frame positions is computed and then divided by the frame interval ($\Delta t$). This captures the magnitude of movement encompassing both *x* and *y* directions. It is ideal for applications where combined horizontal and vertical motion is of interest (e.g., analysing speed across the entire plane of movement).

$$\frac{v\left[i\right] = \sqrt{{\left(x\left[i\right]- x\left[i-1\right]\right)}^{2}+ {\left(y\left[i\right]- y\left[i-1\right]\right)}^{2}}}{\Delta t}$$

In op_compute_velocity(), users can choose whether to compute velocity separately for the two dimensions or merge them into a single metric of velocity, by computing the Euclidean distance (Trettenbrein & Zaccarella, 2021). These options provide flexibility for exploring a range of motion patterns, from simple one-dimensional shifts to complex, multi-dimensional trajectories.

The computed velocity values for each keypoint (prefixed with v_; see Table 3) are stored in a new dataframe, which can then be used to derive acceleration using op_compute_acceleration().

**Table 3:** Example of a dataframe after velocity computation

Person	Frame	Region	v_1	v_2	v_3	v_4
Left	2	Body	82.86	0.51	1.68	2.09
Left	3	Body	0.63	0.49	0.67	0.60
Left	4	Body	0.73	1.08	1.08	1.30
Left	5	Body	82.98	1.61	0.13	0.48
Right	2	Body	0.24	0.31	1.11	2.32
Right	3	Body	0.30	0.40	0.94	2.04
Right	4	Body	0.96	1.58	0.62	0.84
Right	5	Body	0.06	0.31	82.71	0.96

Each row corresponds to a single video frame, with columns representing the person (left or right), frame number, region (e.g., body), and computed velocity values (v_1, v_2, v_3, v_4) for specific keypoints. Velocity is calculated as the Euclidean distance travelled by a keypoint between consecutive frames. The resulting velocity values are expressed in pixels per second

Acceleration represents the rate of change in velocity over time, providing insight into how movement accelerates, decelerates, or shifts direction. The function calculates acceleration for each keypoint based on the velocity data, applying either a single-axis or two-dimensional approach:

$$a\left[i\right]= \frac{v[i] - v[i-1]}{\Delta t}$$

where  $v[i]$ and  $v[i-1]$  are the velocities at consecutive frames, and  $\Delta t$ is the time interval between frames.

$$a\left[i\right]= \frac{\sqrt{{\left({v}_{\chi }[i]- {v}_{\chi }\left[i-1\right]\right)}^{2}+ {\left({v}_{y}\left[i\right]- {v}_{y}\left[i-1\right]\right)}^{2}}}{\Delta t}$$

where  ${v}_{\chi }[i]-$  and ${v}_{y}[i]-$  represent the *x* and *y* velocity components, respectively, at the frame $i$. This measure captures the overall magnitude of acceleration in both dimensions. This will create a new column or each keypoint with the prefix ‘a_’.

The final transformation available is jerkiness or smoothness of movement, which quantifies the rate of change in acceleration over time and provides insight into the smoothness or abruptness of movement. Higher jerk values indicate rapid changes in acceleration, often associated with erratic or unstable motion, while lower values suggest smoother transitions. This metric is particularly relevant in studies examining motor control, coordination, and movement fluidity (Cook et al., 2013; Miller et al., 2023; Schneider & Zernicke, 1989). For a single dimension it can be computed as

$$j\left[i\right]= \frac{a[i] - a[i-1]}{\Delta t}$$

where  $a[i]$  and  $a[-i]$  are the acceleration values at consecutive frames, and  $\Delta t$ is the frame duration. This formulation captures changes in acceleration along a single axis.

$$j\left[i\right]= \frac{\sqrt{{\left({a}_{\chi }[i]- {a}_{\chi }\left[i-1\right]\right)}^{2}+ {\left({a}_{y}\left[i\right]- {a}_{y}\left[i-1\right]\right)}^{2}}}{\Delta t}$$

where  ${a}_{\chi }[i]$  and  ${a}_{y}\left[i\right]$ represent the x and y components of acceleration at frame $i$. This measure captures the magnitude of jerk across both dimensions, reflecting overall movement smoothness in a new column for each keypoint with the prefix ‘j_’.

An alternative to analysing individual kinematic measures is the study of behavioural (motor) synchronisation (Efthimiou et al., 2025b; Kleinbub & Ramseyer, 2021; Tschacher et al., 2014). Synchronisation provides a single measure reflecting the degree to which individuals’ movements are coupled or coordinated over time. This approach can reveal emergent dyadic dynamics that are not apparent from examining individual movements in isolation.

**Fig. 5:**
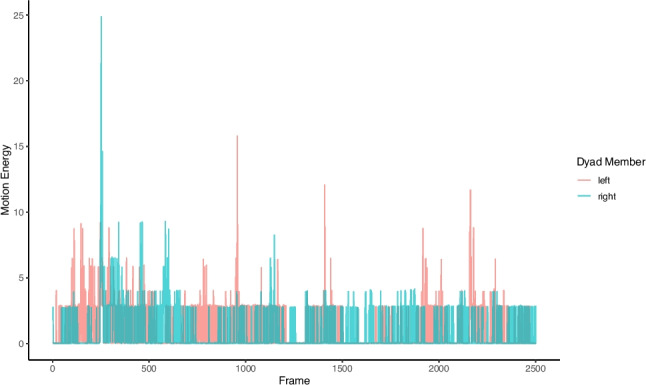
Time series of motion energy for a dyad. *Note*: The plot shows the calculated motion energy for two individuals (left and right person) across the video frames of the interaction. The *y*-axis represents the magnitude of movement, where peaks indicate moments of high activity

To quantify movement, a common preliminary step is to compute a ‘motion energy’, a time series of motion for each individual. This reduces the complex, multi-dimensional raw motion capture data from OpenPose keypoints into a single, continuous variable representing the overall amount of movement for a given body region or the whole body (see Fig. 5).

The op_compute_motionenergy() function uses a technique called frame differencing, in which movement is the change in position between one frame and the next. For any given keypoint, the function calculates how far it has moved from its position in the previous frame to its position in the current frame for both the *y* and *x* axis:

$$\Delta x=xt-xt-1$$

$$\Delta y=yt-yt-1$$

where $\Delta x$ and $\Delta y$ represent the change in *x* and *y* coordinates, respectively, and indicate the distance the point has moved up or down between the two frames. These separate *x* and *y* components of motion can then be combined into a single magnitude for each keypoint using the Euclidean distance. To create a holistic measure of movement, these individual keypoint motions are further aggregated by summing them across the entire body. The function offers two methods for this calculation: the absolute difference, which provides a linear measure of all movement, or the squared difference, which more heavily weights larger, more energetic motions. Finally, if the plot parameter is set to TRUE, the function automatically generates a time-series plot of the aggregated motion energy (as shown in Fig. 5).

```R.

op_compute_motionenergy(df_clean,

method = 'absolute',

aggregate_coordinates = TRUE,

aggregate_keypoints = FALSE,

plot = TRUE).

```

**Fig. 6:**
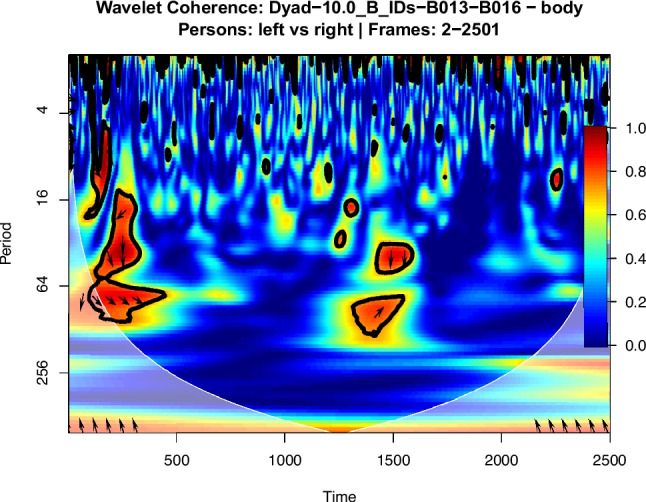
Cross-wavelet coherence plot of motion energy for a single dyad. *Note*: The plot visualises the coherence between the ‘left’ and ‘right’ participants’ motion energy from frames 2 to 2,501. The *x*-axis represents time in video frames, while the *y*-axis shows the period of the movement’s rhythm in frames (where longer periods correspond to slower frequencies). The colour intensity indicates the strength of coherence from low (blue) to high (red), with the colour bar on the right indicating the scale from 0 to 1. Regions enclosed by a thick black contour are statistically significant (*p* <.05). Arrows indicate the phase relationship: right-pointing arrows show in-phase synchrony (moving together), while left-pointing arrows show anti-phase synchrony (mirroring). The area outside the white parabolic curve is the cone of influence (COI), where results should be interpreted with caution due to edge effects

While motion energy gives us a measure of how much each person is moving, coherence tells us whether their patterns of movement are related. It takes the two separate motion energy time series (one for each person in the dyad) and calculates a single measure that reflects how coupled or interdependent their movements are. The output from op_motion_energy() can be directly input into the op_compute_coherence() function to calculate synchrony across different frequencies. Specifically, the function first prepares the data by isolating and temporally aligning the motion energy series for the two individuals. These aligned series are then processed using the wtc function from the biwavelet package (Gouhier et al., 2024), which performs the core mathematical transform to identify shared rhythms (frequencies) over time. A key feature of our implementation is that it summarises the resulting complex coherence map into interpretable statistics. It calculates the average coherence within specific, user-defined frequency bands (e.g., ‘slow rhythms’ from 0.1 to 0.5 Hz) (Fujiwara & Yokomitsu, 2021; Fujiwara et al., 2020), allowing for the testing of hypotheses about coordination at different temporal scales. The final output is a dataframe containing the mean coherence for each frequency band, providing a quantitative measure of dyadic synchrony. The function further produces a plot (see Fig. 6).

### Computational efficiency

To ensure duet is practical for everyday use, we evaluated its performance on a standard laptop (MacBook Pro, Apple M3, 16 GB RAM).

Our goal was to confirm that the package runs efficiently without needing specialised, high-performance hardware. We found that converting a typical 5-min (30 FPS, 7,500 JSON files) video from its raw format into data ready for analysis takes 95.88 s (1.36 min).

To test the functions computing interpolation, kinematics, motion energy, and wavelet coherence, we utilised the bench package (Hester & Vaughan, 2025) to precisely measure execution time and memory allocation for each processing step. For a single dyad (2,500 frames at 30 FPS), we found that the entire workflow, from raw data loading through to final coherence analysis, took approximately 126 s (2.1 min). The vast majority of the package’s functions, particularly for data preparation Like keypoint labelling, interpolation, and velocity calculation, are highly optimised and add very Little time to the process, collectively executing in under 1 s. The most computationally intensive function is the wavelet-based coherence analysis (op_compute_coherence), which accounts for nearly all of the processing time and requires significant memory resources. While most functions in the *duet* package are designed to be memory-efficient for projects of any scale, this specific, advanced analysis is a resource-intensive process. We recommend processing single dyads and concatenating the results after to ensure smooth performance on standard hardware. In summary, *duet* is designed to be fast and responsive for most typical research workflows. For users interested in detailed performance metrics or how to optimise for large-scale analyses, the complete, fully reproducible benchmarking code is documented in the package’s GitHub repository (https://github.com/ThemisEfth/duet/tree/main/demo/package_benchmarks.rmd).

## Conclusions

In this paper, we have introduced *duet,* an R package designed to facilitate the processing and analysis of OpenPose data. The package is free, open-source, and built to streamline the workflow for researchers working with motion capture data, particularly in the context of dyadic interactions. With functionalities ranging from data cleaning, interpolation, and smoothing to kinematic analysis and wavelet coherence, *duet* provides a comprehensive toolkit to prepare OpenPose outputs for advanced analyses. Its user-friendly design and modular structure make it accessible to both novice and experienced users in the fields of psychology, biomechanics, and social interaction research.

Currently, *duet* is specifically designed to work with OpenPose outputs. While we recognise the increasing variety of pose estimation tools available, the present version of duet, including its code, tests, and examples, is tailored to this OpenPose-specific format. The underlying data handling structures within duet have been developed with a general approach to time-series kinematic data, which may facilitate future considerations for broader compatibility should the package evolve. By making *duet* freely available as an open-source R package, we aim to contribute a useful tool to the research community and hope it fosters further exploration and development in motion analysis methodologies.

## Data Availability

The data and materials used in this study are available and can be accessed at https://github.com/ThemisEfth/duet.

## Code availability

The code used in this study is available and can be accessed at https://github.com/ThemisEfth/duet.

## Declarations

### Open Practices Statement

The data and materials for all experiments are available at (https://github.com/ThemisEfth/duet). The paper was not preregistered.
